# The applications of polysaccharides in dentistry

**DOI:** 10.3389/fbioe.2022.970041

**Published:** 2022-07-22

**Authors:** Zhijing Yang, Weiwei Liu, Huimin Liu, Rong Li, Lu Chang, Shaoning Kan, Ming Hao, Dongxu Wang

**Affiliations:** ^1^ Department of Oral and Maxillofacial Surgery, Hospital of Stomatology, Jilin University, Changchun, China; ^2^ Laboratory Animal Center, College of Animal Science, Jilin University, Changchun, China; ^3^ Jilin Provincial Key Laboratory of Tooth Development and Bone Remodeling, Hospital of Stomatology, Jilin University, Changchun, China

**Keywords:** natural polysaccharide, oral disease, tissue repair, tissue regeneration, biomaterials

## Abstract

Polysaccharides are natural polymers widely present in animals, plants, and several microorganisms. Polysaccharides have remarkable properties, including easy extractions, degradability, and renewability, and have no apparent toxicity, making them ideal for biomedical applications. Moreover, polysaccharides are suitable for repairing oral tissue defects and treating oral diseases due to their excellent biocompatibility, biosafety, anti-inflammatory, and antibacterial properties. The oral cavity is a relatively complex environment vulnerable to numerous conditions, including soft tissue diseases, hard tissue disorders, and as well as soft and hard tissue diseases, all of which are complex to treat. In this article, we reviewed different structures of natural polysaccharides with high commercial values and their applications in treating various oral disease, such as drug delivery, tissue regeneration, material modification, and tissue repair.

## Introduction

Oral diseases are a global public health concern, often affecting the aesthetics and diet of patients, and in some cases, they cause systematic diseases (P aradowska-Stolarz et al., 2021; Wong et al., 2019). Treating oral diseases require many biological materials. Dental caries treatment requires filling the dental pulp with resin (Malkondu et al., 2014), pulp capping agent (Kunert and Lukomska-Szymanska, 2020), and gutta percha (Del Fabbro et al., 2016). Also, denture restoration, impression, and dental implant materials are required for tooth loss treatment (Roumanas, 2009). Tissue-guided regeneration and defect repair materials are necessary for oral surgery (Frassica and Grunlan, 2020; Niu et al., 2021). Toothpaste, floss, and mouthwash are needed to prevent oral diseases (Zhang et al., 2021). The common materials used in dentistry include polymethylmethacrylate (PMMA), vulcanized rubber, celluloid, phenol-formaldehyde, and polyvinyl chloride (PVC) (P aradowska-Stolarz et al., 2021). Ideal dentistry materials should possess high biocompatibility as well as good biofunction and mechanical properties (Abdelhamid and Mathew, 2022). In stomatology, natural polysaccharides are used to modify materials repairing chewing organs, and improve oral health (Vergnes and Mazevet, 2020).

Polysaccharides are natural branched or non-branched polymers, usually composed of more than 10 monosaccharides joined together *via* glycosidic bonds (Yu et al., 2018). Polysaccharides are widely present in plants, microorganisms and animals. They possess anti-tumor, anti-oxidation, and anti-inflammatory properties, favouring their application in biomedicine (Silva et al., 2021). In recent years, natural polysaccharides and their derivatives have been widely used in packaging, food and pharmaceutical industries, and dentistry, given their sustainability, renewability, biodegradability, and non-toxicity. Also, the wide application of polysaccharides in the above-mentioned industries is attributed to their structural diversity (Torres et al., 2019). Materials used in the oral cavity are in direct contact with human tissue; thus, they should be biocompatible and non-toxic. Moreover, they should promote efficient healing of the mouth to perform its main functions, including chewing, breathing and talking (Whitters et al., 1999). In addition, given the role of the mouth on the facial outlook, the materials used in the oral cavity need special consideration. Previous reports have shown that natural polysaccharides have numerous advantages in treating oral diseases. In this review, we discussed several natural polysaccharides with high commercial values and their applications in treating different oral diseases.

## Classification of polysaccharides

**FIGURE 1:**
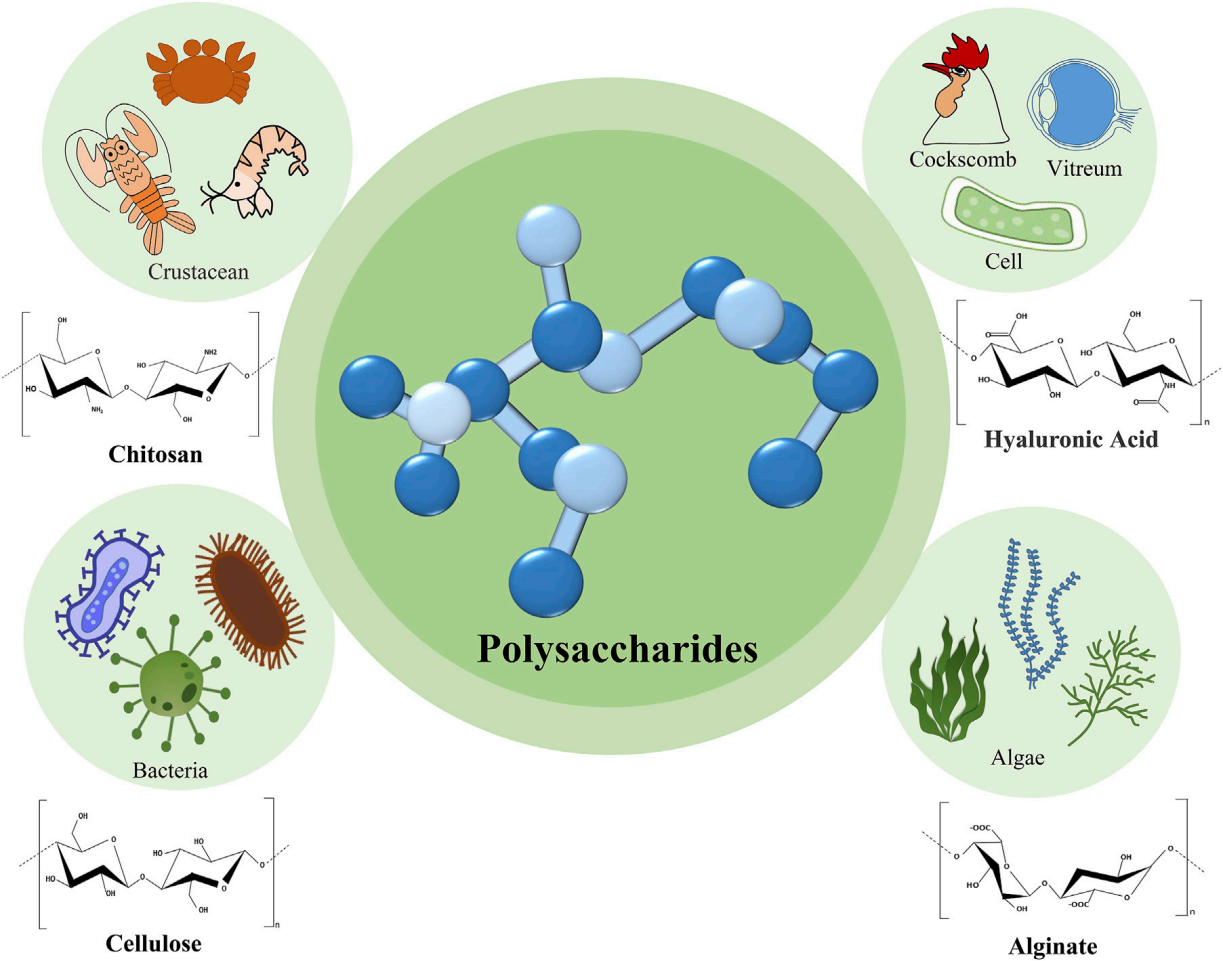
The sources and structures of the commonly used polysaccharides.

Polysaccharides can be broadly classified into homogeneous and heterogeneous polysaccharides. Homogeneous polysaccharides are derived from joining several monosaccharide molecules of the same kind, including starch, cellulose, and chitosan. Heterogeneous polysaccharides are derivatives of different monosaccharide molecules. The common heterogeneous polysaccharides include hyaluronic acid and chondroitin sulfate. Since they are naturally available, easy to obtain, non-toxic, cheap, biodegradable, and biocompatible, polysaccharides are suitable for the oral cavity environment (Prajapati et al., 2014). The most commonly used polysaccharides are shown in Figure 1.

### Chitosan

**FIGURE 2:**
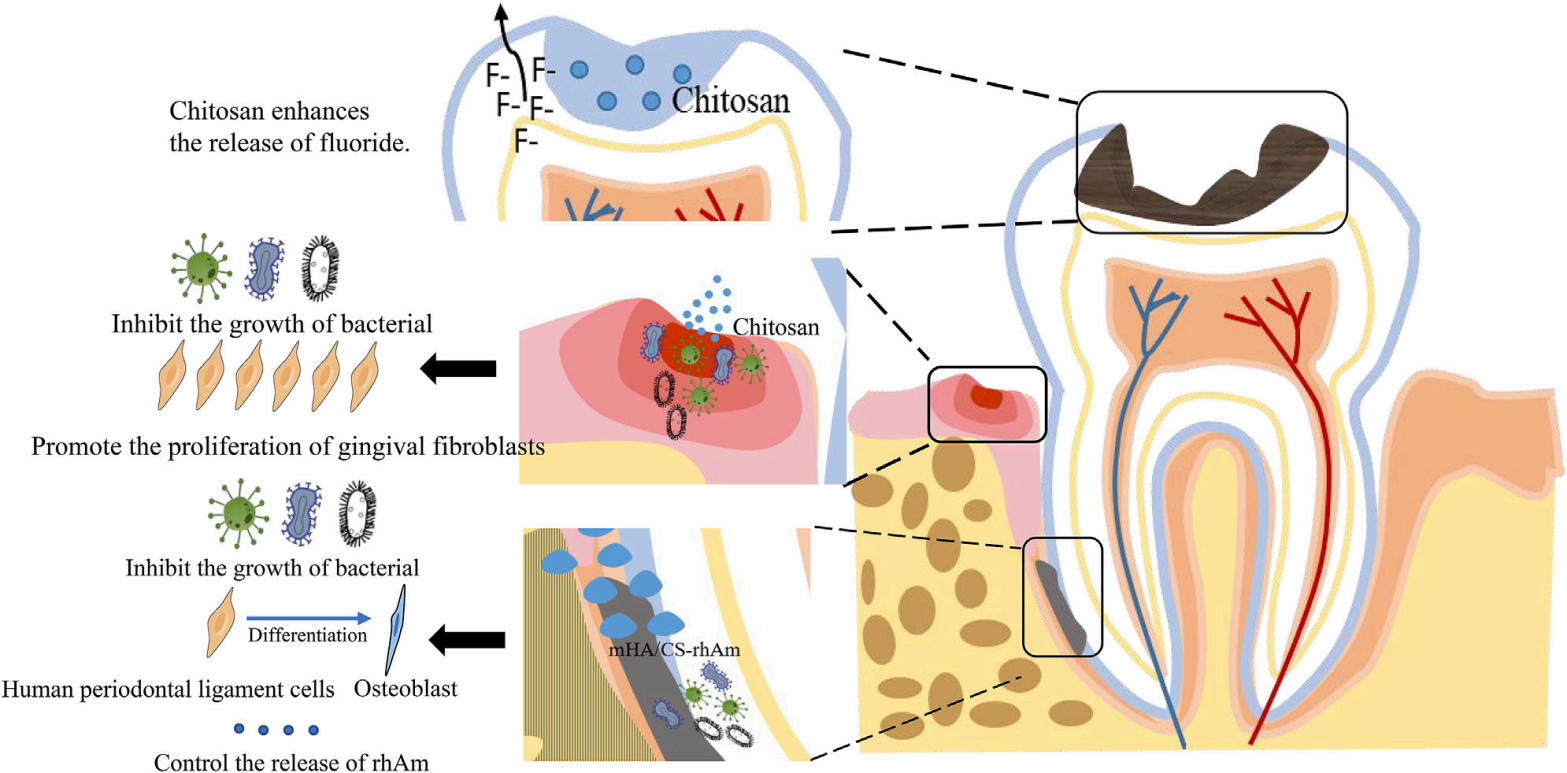
The applications of chitosan in dentistry.

Chitosan is a linear, semi-crystalline natural polysaccharide derived from chitin, readily available in crustaceans and fungal cell walls. The U.S. Food and Drug Administration (FDA) has approved chitosan for food and drug use (Wang et al., 2020). Chitosan contains random glucosamine (deacetylation unit) and N-acetylglucosamine (acetyl unit) units linked to the primary chain through *ß* (1–4) glycosidic bonds. Chitosan is positively charged due to the presence of the amino group (Abd El-Hack et al., 2020). The molecule has a straight-chain structure, and weighs between 10 and 1,000 kDa (Kou et al., 2021). Chitosan is widely utilized for antibacterial purposes (Muxika et al., 2017), drug release (Hudson and Margaritis, 2014), target therapy (Mushtaq et al., 2021; Narmani and Jafari, 2021), and drug delivery in stomatology (Ahsan et al., 2018). Studies have shown that it effectively inhibits the biofilm formation and the acid production capacity of *Streptococcus mutans*, the main causal agent of dental caries (Kawakita et al., 2019). Several delivery systems that carry drugs for anti-inflammatory purposes, tissue, and periodontitis treatment have been modeled around chitosan (Sah et al., 2019; Baranov et al., 2021). Chitosan has been used for modifying nanomaterials and hydrogel, and its derivatives for treating oral diseases have been gradually developed (Wang et al., 2020; Shivakumar et al., 2021) (Figure 2).

### Cellulose

Cellulose is one of the most abundant biological polymers on Earth (Lynd et al., 2002). Plant cellulose is present in most green plants and algae. Bacterial cellulose is naturally secreted by several non-pathogenic bacteria, including *acetobacter*, agrobacterium, and rhizobium. Cellulose has a well-arranged three-dimensional fiber network, 3.0–3.5 μm thick (Klemm et al., 2005; Svensson et al., 2005). Compared with plant cellulose, bacterial cellulose, excluding lignin and hemicellulose, contains small fibers (100 times lower than plant cellulose) and has a highly crystalline structure. Heat, steam, ethylene oxide gas, and radiation will not destroy the inherent physicochemical properties and structural integrity of cellulose (de Oliveira Barud et al., 2020). The three-dimensional structure of cellulose is very similar to the extracellular matrix (ECM) of living tissues and, thus, suitable for preserving oxygen and nutrients, providing a favourable environment for the growth and proliferation of cells in stomatology (Halib et al., 2019). Moreover, cellulose can be used for tissue engineering as scaffolds and implants for wound healing, drug delivery, and dental materials (de Oliveira Barud et al., 2016; de Oliveira Barud et al., 2020; Klinthoopthamrong et al., 2020). Studies have shown that restorable BC could be used in GBR to improve bone generation. Moreover, the electron beam irradiation (EI) can sever the BC glucose bonds to increase biodegradation (An et al., 2017).

### Hyaluronic acid

**FIGURE 3:**
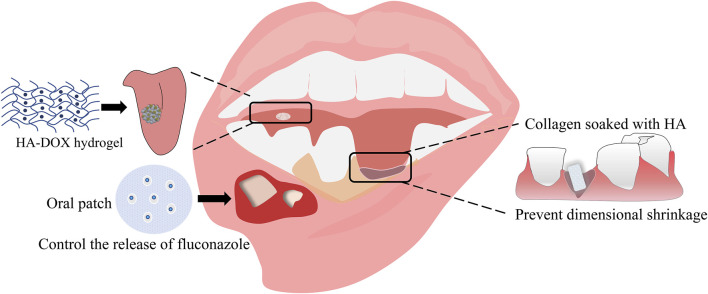
The applications of HA in dentistry.

Hyaluronic acid (HA) is a glycosaminoglycan composed of repeated N-acetyl d-glucosamine and d-glucuronic acid units (Marinho et al., 2021). It is the main skin extracellular matrix (ECM) component and participates in inflammatory responses, angiogenesis, and tissue regeneration. Given its anti-inflammatory property, HA is often used to treat oral ulcers (Graca et al., 2020). HA is also a good drug delivery system, given its mucosal adhesion and ease of chemical modification (Huang and Huang, 2018; Salari et al., 2021). Notably, a local administration strategy is preferred for treating oral diseases than systemic administration. HA does not affect other body organs and tissues when used for drug delivery. Moreover, the local treatment ensures better delivery of drugs to soft periodontal tissue, gingiva, periodontal ligament, and hard tissues, such as alveolar bone and cementum. Catechol (Cat)-modified chitosan/hyaluronic acid nanoparticles (NPs) is a newly developed system for delivering doxorubicin (DOX) to oral squamous cell carcinoma. It displays excellent adhesion to the oral mucosa and delivery of DOX to the target tissues in a sustained manner (Pornpitchanarong et al., 2020). HA has good biocompatibility, biodegradability, and hydrophilicity and can alleviate inflammatory pain. These desired biological properties promote the use of HA in the oral cavity (Figure 3).

### Marine algae polysaccharides

Seaweed polysaccharides, including alginate, agar, carrageenan, galactan, fucoidan, urfan, and laver polysaccharides, are widely used in pharmaceutical industry (Rahmati et al., 2019; Beaumont et al., 2021). Marine algae polysaccharides possess numerous physical and chemical properties. These types of polysaccharides are soft and expansible (Bilal and Iqbal, 2019). Sodium alginate and agarose, widely used in treating oral diseases, are the common marine algae polysaccharides. Sodium alginate was included on the list of Pharmacopoeia products in the United States as early as 1938. Sodium alginate is a by-product of kelp or sargassum, ß-d-mannuronic acid (β-D-mannuronic, m), and *a*- l-guluronic acid (α-L-guluronic, g), joined together *via* the 1 → 4 linkages. The compound is stable, soluble, viscous, and safe, suitable for pharmaceutical use (Sanchez-Ballester et al., 2021). Furthermore, it contains numerous -COO- units that give it its polyanionic behavior in an aqueous solution, facilitating drug attachment and adhesion to the mucosal membrane (Amin and Boateng, 2022). Some studies have suggested that sodium alginate could treat recurrent aphthous ulcers. The compound can alleviate the pain and improve the persistence of a drug in the oral mucosa (Laffleur and Kuppers, 2019; Suharyani et al., 2021). In addition, alginate is mainly used for stomatology as an impression of oral materials (Cervino et al., 2018). Obtaining an oral model is an important step before orthodontics and prosthodontics treatment. Alginate impression materials have remarkable fluidity, resilience, plasticity, and high drug delivery accuracy, promoting their clinical use.

Agarose is a linear polymer polysaccharide extracted from kelp (Zucca et al., 2016), composed of alternating 1→3-linked ß-Dgalactopyranose and 1→4-linked 3,6 anhydro-α-l-galactopyranose units, designated as G and A residues, respectively (Trivedi et al., 2015). Agarose is a potential hydrogel for the controlled release of bioactive substances, given its good biocompatibility and natural biodegradability (Kim et al., 2019). In the oral cavity, agarose gel regulates the size and shape of hydroxyapatite crystals (Ling et al., 2019). A new biomimetic mineralization system containing agarose gel can induce a dense hydroxyapatite layer on the surface of demineralized dentin to block dentin tubules for dentin remineralization, a potentially new method for treating dentin hypersensitivity and dental caries (Ning et al., 2012). These two algal polysaccharides are highly biocompatible and have broad dental applications, including treating oral diseases.

## Applications of natural polymers in stomatology

**FIGURE 4:**
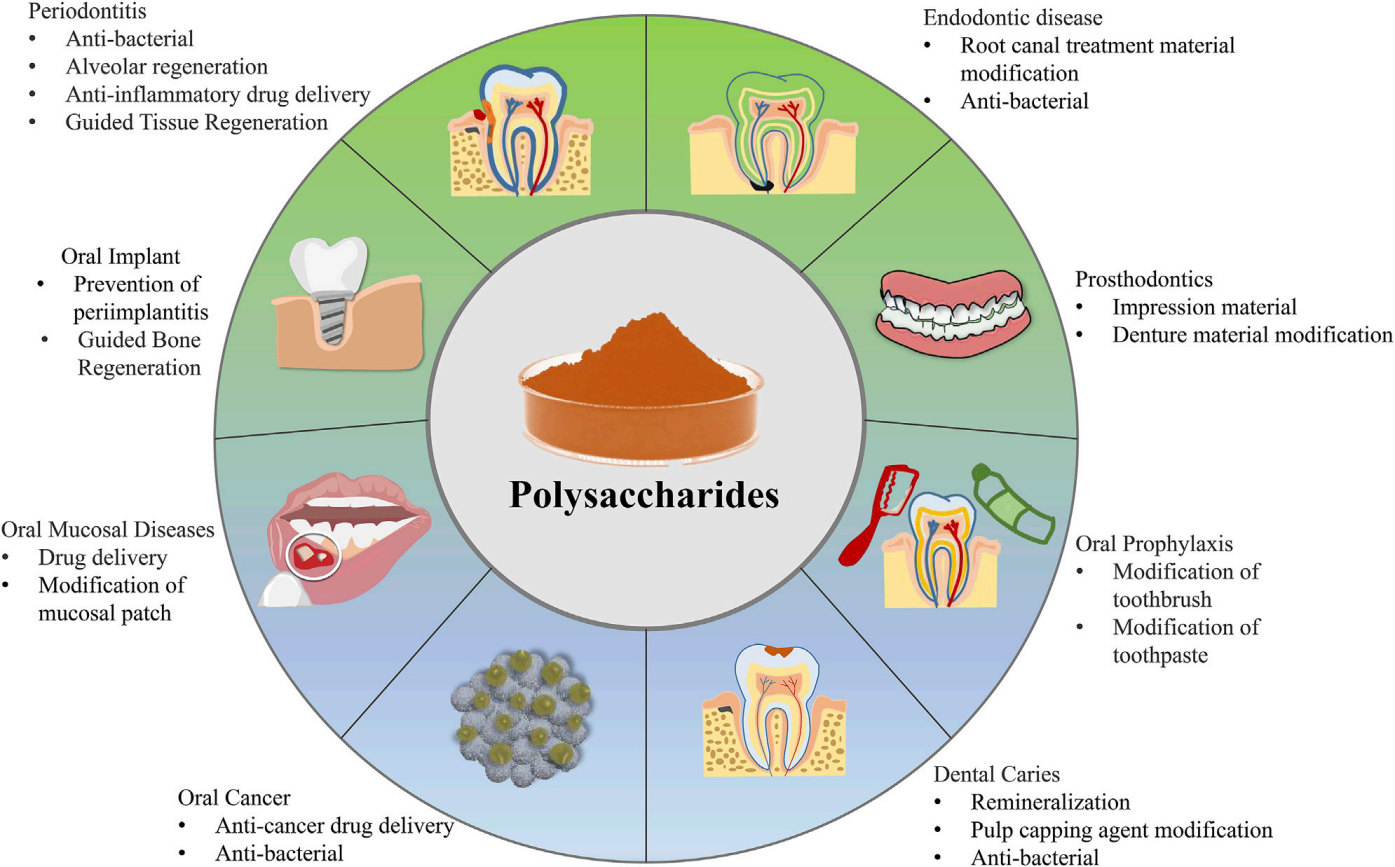
The applications of polysaccharides in dentistry.

The oral cavity is located in the lower 1/3 of the maxillofacial region, starting from the lips. It has several sections, including the buccal, the inner lining of the cheeks, the pharynx at the back, the palate on the upper side, and the floor of the mouth on the lower side. Together, these parts form the cavitas oris propria (Tuominen and Rautava, 2021). Dental caries is an idiopathic disease of the oral cavity (Akintoye and Mupparapu, 2020). The teeth structure is different from that of bones and includes the enamel, dentin, pulp, and cementum. Enamel is the hardest tissue in the body that has neither blood vessels nor nerves and cannot regenerate. The dentin is softer than the enamel and can regenerate or repair itself to a certain degree. However, excessive teeth damage can create irreversible defects. If dental caries is not treated on time, the enamel is continually eaten up, causing bacteria infection in the pulp and periapical tissue. This eventually causes pulpitis, periapical periodontitis, jaw osteomyelitis, and other concurrent diseases, inducing severe pain and tissue damage. Moreover, dental caries not only destroy the integrity of the chewing organs but also affects the digestive function, seriously affecting an individual’s overall health. Natural polysaccharides, which have antibacterial, drug delivery, and material modification capability with remarkable biocompatibility, are suitable for different dental functions to prevent oral diseases of soft and hard tissues or a combination of both (Figure 4).

### Treatment of oral hard tissue diseases

Oral hard tissue diseases include dental caries, maxillofacial fractures, and bone defects (Melo et al., 2013). In dentistry, chitosan and alginate are used to improve the property of adhesive material. A combination of chitosan and triclosan supplemented with resin inhibits biofilm formation and improves the stability between dentin and adhesive interface, promoting long-term edge sealing (Machado et al., 2019). A mixture of chitosan and glass ionomer cement (GIC) can be another repair material that allows the slow-release of fluoride (Ibrahim et al., 2020). Chitosan reacts with the GIC to generate a compound with a better ion release rate, which is important for tooth structure (Mulder and Anderson-Small, 2019). Moreover, a formulation of type I collagen (Col), nanocrystalline hydroxyapatite (HAp), and alginate (Alg) is suitable for 3D printing of scaffolds with properties similar to those of natural dentin. The formulation effectively treats tooth sensitivity by blocking the dentin microtubules (Naseri et al., 2021). On the whole, polysaccharides could be modified to generate desired products suitable for treating hard tissue diseases in dentistry.

### Treatment of oral soft tissue diseases

Diseases of the soft mouth tissues include pulpitis, periapical periodontitis, gingivitis, and other oral mucosal diseases. Infected dental pulp is very painful, significantly affecting the normal life of patients. The infection can spread to periapical tissues, causing periapical periodontitis. Gingivitis is an inflammation of teeth gums caused by accumulated dental plaque and some physical stimulation in the papillary. The disease can cause periodontitis and loss of the alveolar bone. Therefore, removing the root canal infection and dental plaque is important in treating oral soft tissue diseases. Given the unique anatomy, histology, and microbial environment of the oral cavity, materials for oral cavity uses should possess anti-bacterial properties, adhere on the oral cavity, be easy to apply, and should allow slow release of important molecules (Karolewicz, 2016; Timur et al., 2019). Natural polysaccharides can be modified to possess good adhesion, repair, and mechanical properties suitable for treating oral soft tissue diseases.

Chitosan inhibits the growth of *Porphyromonas gingivalis* and *Actinomyces aggregatum* and can modulate inflammation in human gingival fibroblasts by regulating the level of PGE2 through the JNK pathway. Chitosan is well tolerated by gingival fibroblasts and can stimulate cell proliferation via the ERK1/2 signaling pathway. Furthermore, the synergistic effect of chitosan and growth factors such as PDGF-BB stimulates the proliferation of gingival fibroblasts and inhibits the growth of *Porphyromonas gingivalis* and *Actinomyces aggregatum* (Silva et al., 2013). Bacterial cellulose fastens the solidification of diatomite and enhances the biological activity of the mineral. Moreover, it has good biocompatibility and promotes the proliferation and adhesion of mesenchymal stem cells (Voicu et al., 2017). In addition, it does not generate oxidative stress and is, therefore, an excellent material in endodontics. Compared with the ordinary absorbent paper tips, bacterial cellulose has a higher moisture absorption rate, expansion rate, tensile strength, and biocompatibility. Moreover, it is hard and causes no obvious allergic reactions (Yoshino et al., 2013). HA and platelet lysates’ complex increase the metabolism of dental pulp mesenchymal stem cells and repair damaged dental pulp/dentin tissue by stimulating the deposition of mineralized matrix (Almeida et al., 2018; Schmidt et al., 2020). A combination of bacterial cellulose (BC) and photoactivated carbene-based biological adhesive (PDZ) forms a flexible film platform that can repair soft tissue in the ever-wet mouth environment. The shear strength and adhesion of the composite have been significantly improved, making it suitable for treating oral mucosal wounds (Singh et al., 2021). Therefore, polysaccharides can be used as antibacterial, root canal therapy materials and tissue treating oral patches, underlining the novel application prospects of these materials.

### Treatment of a combination of oral soft and hard tissue diseases

Diseases involving soft and hard tissues in the mouth include cancer and periodontitis. Both diseases can cause lesions, gingival, buccal and lingual mucosa recession, and even alveolar bone and jaw bone loss. Gingival recession and the alveolar bone defect caused by periodontitis are irreversible and greatly affect a person’s facial appearance. In serious cases, it causes tooth loss and facial deformity, affecting eating and talking. Therefore, periodontitis should be promptly treated. The periodontium repair involves using multiple materials to prevent further damage and restore bone and periodontal losses. Pure chitosan scaffolds promote the proliferation of cementoblasts (CB) and periodontal ligament cells (PDLCs), the alkaline phosphatase activity, and mineralization level. The CS scaffolds, combined with other polymer biomaterials and bioceramics, promote rapid periodontal regeneration (Lauritano et al., 2020). Mesoporous hydroxyapatite/chitosan (MHA/CS) composite scaffolds promote periodontal tissue regeneration, inhibit the growth of *Clostridium nucleatum* and *Porphyromonas gingivalis*, promote the differentiation of periodontal ligament stem cells into osteoblasts, and upregulate the ALP activity, and the expression of RUNX-2, OPN, and DLX-5 *in vitro*. Moreover, MHA/CS composite induces the cementum-like tissue formation *in vivo*, demonstrating its potential for periodontal tissue regeneration (Liao et al., 2020). Mice models with bone defects comparing bacterial cellulose and collagen biofilms regarding guided bone regeneration during periodontal tissue repair revealed that bacterial cellulose biofilms only promote soft tissue repair in skulls but do not induce bone regeneration (Farnezi Bassi et al., 2020). However, hydrolyzed cellulose biofilm through strontium apatite modulates inflammation at the wound site and promotes the formation of connective tissue and the increase of calcium and magnesium, important elements that promote the bone generation and calcification (Luz et al., 2020). In general, the natural polysaccharides can effectively induce periodontal tissue regeneration, supporting periodontitis treatment.

Oral cancer is another disease of oral soft and hard tissues. Oral cancer is a life-threatening disease, whether local or metastatic. Natural polysaccharides have remarkable adhesion property, which is suitable for the wet oral environment. The polysaccharide sticks to the oral tissues, ensuring precise and sustained delivery of antibiotics. Combining bacterial cellulose, alginate, gelatin, and curcumin forms a multifunctional biopolymer film material that can release curcumin in saliva and has no obvious toxicity to human keratinocytes and human gingival fibroblasts. However, the biopolymer inhibits the growth of oral cancer cells and has good antibacterial activity against *Escherichia coli* and *Staphylococcus aureus* and, thus, is suitable for topical wound care and periodontitis and oral cancer treatment (Chiaoprakobkij et al., 2020). TQ/Ca-alg-PVA, a product of loading with calcium alginate and polyvinyl alcohol onto thymoquinone (TQ), inhibits early-stage oral cancer in 7,12-Dimethylbenz [a]anthracene (DMBA) painted hamster by downregulating the expression of NF-κB p50/p65, and PI3K/AKT/mTOR mRNA (Pu et al., 2021). In summary, natural polysaccharides can achieve precise treatment and reduce the drug resistance of oral cancer.

### Oral care

Caries and periodontal disease, the most common disease in the oral cavity, is primarily prevented through oral hygiene. Mouthwash containing natural polysaccharides such as chitosan is a safe and effective natural product for reducing harmful oral microorganisms (Farias et al., 2019). Chitosan mouthwash has been proven safe, and its cytotoxicity is lower than that of commercial mouthwash, and effectively inhibits *Streptococcus* spp. *And Enterococcus spp*, preventing oral diseases (Costa et al., 2014). Paper-like nanofiber materials made from bacterial cellulose and chitosan inhibit the growth of bacteria and yeast, biofilm formation, and oxidation (Cabanas-Romero et al., 2020). Given that polysaccharides are biodegradable, they are environmentally friendly biomaterials. Chitosan-based toothpaste prevents tooth enamel erosion and wear. Toothpaste supplemented with chitosan and Sn^2+^ prevents corrosion and abrasion of teeth gums (Schluet er et al., 2014). In addition, chitosan-containing chewing gum reduces enamel demineralization and maintains bacteriostatic levels in saliva (Hayashi et al., 2007). Thus, the polysaccharides can be supplemented in the oral care products to prevent oral diseases.

Alveolar ridge preservation after tooth extraction remains an oral implant challenge. A mixture of DBBM-C and HA covered in a collagen membrane prevents dimensional shrinkage and increases bone formation after tooth extraction (Lee et al., 2021). Colonization of the bacteria is a major cause of implant failure (Jiang et al., 2020). Studies have shown that chitosan reduces colonization of *Fusobacterium nucleatum* on the implant surface, plaque biofilm formation, and decrease periimplantitis, increasing the success of implanting (Vaz et al., 2018). Therefore, chitosan is a remarkable antibacterial material that can improve the success of oral implants. Generally, the natural polysaccharide has good biocompatibility and is non-toxic and, thus, an attractive oral care material in stomatology.

## Discussion

In recent years, natural polysaccharides extracted from animals, plants, and microorganisms have attracted the attention of researchers because of their good degradability, non-toxicity, and renewability (Zhao et al., 2020). At present, the antibacterial, anti-inflammatory, modifiable, tissue regeneration, and drug carrier potential of polysaccharides have been investigated (Cui et al., 2018; Serrano-Sevilla et al., 2019; Hou et al., 2020; Layek and Mandal, 2020; Zhai et al., 2020). Different polysaccharides are used for treating different oral diseases (Table 1). Natural polysaccharides have attracted greater attention than synthetic materials because they are biocompatible, biodegradable, and ecological.

**TABLE 1:** The applications of polysaccharide in oral diseases.

Polysaccharides	Dental Specialties	Models	Biological Activity/Application	References
Chitosan dispersion	Dental caries	*In vitro*	Exert antimicrobial effect against mature S. mutans biofilms	Kawakita et al. (2019)
Bacterial cellulose	Bone regeneration	*In vivo* rat calvarial defect models	The BC biofilms exhibited significantly larger new bone area *in vivo*	Coelho et al. (2019)
Hyaluronic acid	Oral squamous cell carcinoma	*Ex vivo* porcine oral mucosal tissues	Deliver DOX to HN22 with a low IC50	Pornpitchanarong et al. (2020)
Hyaluronic acid	Oral candidiasis	*In vivo* sheep buccal mucosa	Hyaluronic acid hydrogel delivers a nanotransfersome with fluconazole entrapped, which exert enhanced antifungal efficacy	Alkhalidi et al. (2020)
Alginate	Recurrent aphthous stomatitis	*In vitro*	Adhesion time was improved and the AL. Ambroxol was controlled release	Laffleur and Kuppers (2019)
Alginate	Tooth sensitivity	*In vitro*	The 3D printing dentin mimics is of good cytocompability and could block the dentinal tubule effectively	Naseri et al. (2021)
Bacterial cellulose	Pulpitis	*In vivo* Sprague–Dawley rat	BC showed higher absorption and expansion than paper points, and maintained a high tensile strength even wet.	Yoshino et al. (2013)
Chitosan	Periodontitis	*In vitro*	Chitosan induces the proliferation of human gingival fibroblasts by activating of the ERK1/2 signaling pathway	Silva et al. (2013)
Agarose	Dentin hypersensitivity and dental caries	*In vitro*	Induced a layer of tightly packed hydroxyapatite on the surface of demineralized dentine and the dentinal tubules was occluded	Ning et al. (2012)
Chitosan	Mouthwash	*In vitro*	The chitosan mouthwash inhibits the streptococci and enterococci and cause no major reductions to the normal oral microflora viability	Costa et al. (2014)
Chitosan	Dental caries filling materials	*In vitro*	Triclosan-loaded chitosan showed antibacterial activity and induced dentin/adhesive interface stability	Machado et al. (2019)
Chitosan	Modify glass ionomer restorative cements	*In vitro*	Chitosan modifications increase the ion release of aluminium, sodium, silicon and strontium for materials	Mulder and Anderson-Small (2019)
Chitosan	The nano hydroxyapatite/chitosan composite scaffold for periodontal regeneration	*In vivo*	mHA/CS could promote periodontal regeneration	Liao et al. (2020)
Bacterial cellulose	The guided bone regeneration (GBR) membranes	BALB/c mice	Promoting soft tissue repair in rat skulls	Farnezi Bassi et al. (2020)
Bacterial cellulose	The guided bone regeneration (GBR) membranes	*In vivo*	Modulates inflammation, promotes the formation of connective tissue and the increase of calcium and magnesium	Luz et al. (2020)
Bacterial cellulose/Alginate	Oral mucoadhesive patches for periodontitis or oral cancer treatment	Rats	Showing anticancer activity against oral cancer cells (CAL-27), but non-cytotoxicity to HaCaT and GF cells	Chiaoprakobkij et al. (2020)
Alginate	Oral cancer	*In vivo*	Inhibits early-stage oral cancer	Pu et al. (2021)
Chitosan	Mouthwash	Swiss albino mice	Antimicrobial effectiveness and toxicological safety	Farias et al. (2019)
Chitosan	Toothpaste	*In vitro* porcine mucosa	Chitosan enhanced the efficacy of the Sn^2+^-containing toothpaste as an anti-erosive/anti-abrasive agent	Schlueter et al. (2014)
Chitosan	Chewing gum	*In vivo* hamster buccal	Chitosan-containing gum chewing has a better antibacterial effect and increases salivary secretion	Hayashi et al. (2007)
Hyaluronic Acid	Ridge preservation	*In vitro*	Prevents dimensional shrinkage and increases bone formation after tooth extraction	Lee et al. (2021)

Despite the advantages of natural polysaccharides in dentistry, these molecules have certain disadvantages in biomedical applications; 1) They have poor mechanical properties. The inferior adhesiveness and the short-term *in vivo* stability of natural polysaccharides limit their therapeutic efficacy (Kim et al., 2021). 2) The quality of polysaccharides is limited to their original material. The method of extraction and purification affects controlled the products’ reliability (Luo et al., 2021). 3) Natural polysaccharides are highly moisture sensitive. They undergo hydrolysis during processing and are unstable in the oral cavity (Imre et al., 2019).

Natural polysaccharides are generated through natural processes. However, researches are needed to explore strategies for modifying the chemical structure of these molecules to broaden their biomedical applications. Moreover, the natural polysaccharides are made to the namomaterials (Georgouvelas et al., 2021), which might improve their inherent properties to enhance drug delivery efficacy (Ahmed and Aljaeid, 2016) and applied in wound healing (Yang et al., 2020). In future, natural material could be brought to clinical practice and their effect to environment should be valued. It is believed that advances in the development of biomaterials and molecular biology-related technologies will enhance the application of natural polysaccharides in stomatology.
